# Supplementary material for: Modeling glioblastoma heterogeneity as a dynamic network of cell states
Source: Mol Syst Biol. 2021 Sep 16;17(9):e10105. doi: 10.15252/msb.202010105 (PMC8444284; doi:10.15252/msb.202010105)
Supplement: Supplementary file 5 — Source Data for Figure 3 [file MSB-17-e10105-s001.zip › Figure3A_sourcedata/GSEA_3065/nosigdb_state4.GseaPreranked.1622718430994/SEURAT_CELLCYCLE.html]

Details for gene set SEURAT\_CELLCYCLE[GSEA]

|  || Dataset | state4 |
| Phenotype | NoPhenotypeAvailable |
| Upregulated in class | na\_neg |
| GeneSet | SEURAT\_CELLCYCLE |
| Enrichment Score (ES) | -0.8615082 |
| Normalized Enrichment Score (NES) | -3.068298 |
| Nominal p-value | 0.0 |
| FDR q-value | 0.0 |
| FWER p-Value | 0.0 |
Table: GSEA Results Summary

  

Fig 1: Enrichment plot: SEURAT\_CELLCYCLE      
 Profile of the Running ES Score & Positions of GeneSet Members on the Rank Ordered List

  

| PROBE | GENE SYMBOL | GENE\_TITLE | RANK IN GENE LIST | RANK METRIC SCORE | RUNNING ES | CORE ENRICHMENT || 1 | PSRC1 |  |  | 2990 | 0.025 | -0.4834 | No |
| 2 | CBX5 |  |  | 3561 | 0.010 | -0.5754 | No |
| 3 | G2E3 |  |  | 4722 | -0.062 | -0.7612 | No |
| 4 | POLA1 |  |  | 5058 | -0.088 | -0.8124 | No |
| 5 | TIPIN |  |  | 5131 | -0.095 | -0.8208 | No |
| 6 | DSCC1 |  |  | 5133 | -0.095 | -0.8178 | No |
| 7 | UNG |  |  | 5148 | -0.096 | -0.8167 | No |
| 8 | GAS2L3 |  |  | 5152 | -0.097 | -0.8139 | No |
| 9 | UBR7 |  |  | 5340 | -0.112 | -0.8403 | No |
| 10 | RPA2 |  |  | 5372 | -0.115 | -0.8414 | No |
| 11 | WDR76 |  |  | 5388 | -0.117 | -0.8398 | No |
| 12 | EXO1 |  |  | 5410 | -0.120 | -0.8391 | No |
| 13 | RANGAP1 |  |  | 5463 | -0.127 | -0.8432 | No |
| 14 | NEK2 |  |  | 5505 | -0.132 | -0.8453 | No |
| 15 | CKAP5 |  |  | 5549 | -0.136 | -0.8476 | No |
| 16 | PRIM1 |  |  | 5636 | -0.150 | -0.8564 | Yes |
| 17 | CDCA8 |  |  | 5642 | -0.151 | -0.8520 | Yes |
| 18 | MCM6 |  |  | 5644 | -0.151 | -0.8470 | Yes |
| 19 | CASP8AP2 |  |  | 5646 | -0.152 | -0.8419 | Yes |
| 20 | TTK |  |  | 5684 | -0.157 | -0.8425 | Yes |
| 21 | MSH2 |  |  | 5711 | -0.162 | -0.8412 | Yes |
| 22 | E2F8 |  |  | 5717 | -0.163 | -0.8364 | Yes |
| 23 | SLBP |  |  | 5748 | -0.172 | -0.8354 | Yes |
| 24 | BRIP1 |  |  | 5769 | -0.177 | -0.8326 | Yes |
| 25 | NCAPD2 |  |  | 5778 | -0.179 | -0.8277 | Yes |
| 26 | MCM2 |  |  | 5785 | -0.180 | -0.8225 | Yes |
| 27 | CCNE2 |  |  | 5827 | -0.190 | -0.8227 | Yes |
| 28 | BLM |  |  | 5833 | -0.191 | -0.8169 | Yes |
| 29 | CDCA7 |  |  | 5863 | -0.198 | -0.8149 | Yes |
| 30 | CKAP2 |  |  | 5879 | -0.201 | -0.8104 | Yes |
| 31 | POLD3 |  |  | 5957 | -0.225 | -0.8151 | Yes |
| 32 | CENPA |  |  | 5960 | -0.226 | -0.8077 | Yes |
| 33 | DTL |  |  | 5975 | -0.229 | -0.8022 | Yes |
| 34 | USP1 |  |  | 5986 | -0.233 | -0.7958 | Yes |
| 35 | KIF2C |  |  | 5996 | -0.237 | -0.7891 | Yes |
| 36 | AURKA |  |  | 6026 | -0.248 | -0.7853 | Yes |
| 37 | FEN1 |  |  | 6035 | -0.256 | -0.7778 | Yes |
| 38 | BUB1 |  |  | 6045 | -0.266 | -0.7702 | Yes |
| 39 | HJURP |  |  | 6055 | -0.271 | -0.7623 | Yes |
| 40 | CDC45 |  |  | 6063 | -0.276 | -0.7540 | Yes |
| 41 | MCM5 |  |  | 6069 | -0.278 | -0.7453 | Yes |
| 42 | GMNN |  |  | 6080 | -0.285 | -0.7371 | Yes |
| 43 | NUF2 |  |  | 6093 | -0.293 | -0.7290 | Yes |
| 44 | RFC2 |  |  | 6095 | -0.294 | -0.7191 | Yes |
| 45 | CDC6 |  |  | 6103 | -0.299 | -0.7100 | Yes |
| 46 | CKAP2L |  |  | 6108 | -0.303 | -0.7002 | Yes |
| 47 | KIF11 |  |  | 6109 | -0.304 | -0.6898 | Yes |
| 48 | LBR |  |  | 6118 | -0.312 | -0.6804 | Yes |
| 49 | CDCA2 |  |  | 6135 | -0.326 | -0.6718 | Yes |
| 50 | TACC3 |  |  | 6138 | -0.328 | -0.6609 | Yes |
| 51 | CCNB2 |  |  | 6140 | -0.329 | -0.6497 | Yes |
| 52 | NDC80 |  |  | 6142 | -0.330 | -0.6386 | Yes |
| 53 | AURKB |  |  | 6143 | -0.332 | -0.6272 | Yes |
| 54 | RRM1 |  |  | 6147 | -0.338 | -0.6161 | Yes |
| 55 | HMMR |  |  | 6148 | -0.340 | -0.6044 | Yes |
| 56 | CDC20 |  |  | 6154 | -0.344 | -0.5934 | Yes |
| 57 | DLGAP5 |  |  | 6156 | -0.345 | -0.5818 | Yes |
| 58 | KIF23 |  |  | 6162 | -0.351 | -0.5706 | Yes |
| 59 | CDCA3 |  |  | 6165 | -0.355 | -0.5587 | Yes |
| 60 | RAD51AP1 |  |  | 6167 | -0.357 | -0.5467 | Yes |
| 61 | ANP32E |  |  | 6168 | -0.357 | -0.5344 | Yes |
| 62 | MCM4 |  |  | 6171 | -0.372 | -0.5220 | Yes |
| 63 | KIF20B |  |  | 6172 | -0.373 | -0.5092 | Yes |
| 64 | PCNA |  |  | 6173 | -0.374 | -0.4964 | Yes |
| 65 | ECT2 |  |  | 6178 | -0.382 | -0.4839 | Yes |
| 66 | NASP |  |  | 6187 | -0.403 | -0.4714 | Yes |
| 67 | GINS2 |  |  | 6194 | -0.420 | -0.4580 | Yes |
| 68 | TUBB4B |  |  | 6196 | -0.431 | -0.4433 | Yes |
| 69 | UHRF1 |  |  | 6198 | -0.436 | -0.4286 | Yes |
| 70 | RRM2 |  |  | 6199 | -0.438 | -0.4136 | Yes |
| 71 | BIRC5 |  |  | 6214 | -0.484 | -0.3992 | Yes |
| 72 | ANLN |  |  | 6215 | -0.484 | -0.3826 | Yes |
| 73 | GTSE1 |  |  | 6217 | -0.490 | -0.3660 | Yes |
| 74 | CLSPN |  |  | 6218 | -0.490 | -0.3492 | Yes |
| 75 | SMC4 |  |  | 6219 | -0.490 | -0.3324 | Yes |
| 76 | HELLS |  |  | 6221 | -0.501 | -0.3154 | Yes |
| 77 | TYMS |  |  | 6222 | -0.501 | -0.2982 | Yes |
| 78 | NUSAP1 |  |  | 6223 | -0.507 | -0.2808 | Yes |
| 79 | CENPE |  |  | 6226 | -0.511 | -0.2636 | Yes |
| 80 | TMPO |  |  | 6233 | -0.522 | -0.2467 | Yes |
| 81 | CKS1B |  |  | 6234 | -0.523 | -0.2287 | Yes |
| 82 | CKS2 |  |  | 6239 | -0.585 | -0.2093 | Yes |
| 83 | ATAD2 |  |  | 6243 | -0.607 | -0.1890 | Yes |
| 84 | CDK1 |  |  | 6246 | -0.627 | -0.1678 | Yes |
| 85 | TPX2 |  |  | 6253 | -0.667 | -0.1459 | Yes |
| 86 | UBE2C |  |  | 6255 | -0.750 | -0.1204 | Yes |
| 87 | HMGB2 |  |  | 6258 | -0.766 | -0.0944 | Yes |
| 88 | TOP2A |  |  | 6260 | -0.814 | -0.0667 | Yes |
| 89 | MKI67 |  |  | 6261 | -0.882 | -0.0365 | Yes |
| 90 | CENPF |  |  | 6263 | -1.068 | 0.0000 | Yes |
Table: GSEA details [plain text format]

  

Fig 2: SEURAT\_CELLCYCLE: Random ES distribution      
 Gene set null distribution of ES for **SEURAT\_CELLCYCLE**
